# Statistical interval-based assessment of work disability using large-scale medical data: implications for public health and workforce participation

**DOI:** 10.3389/fresc.2026.1851082

**Published:** 2026-09-15

**Authors:** Lyazat M. Aktayeva, Aiman S. Iskakova

**Affiliations:** Republican Research Institute for Occupational Safety and Health, Ministry of Labor and Social Protection of the Population of the Republic of Kazakhstan, Astana, Kazakhstan

**Keywords:** disability assessment, functional impairment, occupational health, work disability, workforce participation

## Abstract

**Objectives:**

Work disability assessment is a key component of occupational health systems, influencing compensation, return-to-work decisions, and workforce management. However, variability in expert evaluations remains a major challenge. This study aims to identify recurring population-level patterns of disability severity using large-scale medical expert data.

**Methods:**

A retrospective dataset of 4,863 disability assessments conducted between 2020 and 2024 was analyzed. After applying inclusion criteria, 2,065 observations across 161 ICD-10 diagnostic categories were included. Statistical analysis involved empirical distribution modeling, concentration function estimation, confidence interval analysis, and hypothesis testing. Robustness was assessed using Monte Carlo resampling. Sensitivity analyses indicated that the predefined intervals were frequently reproduced as dominant concentration intervals under repeated resampling.

**Results:**

Disability severity showed distinct concentration patterns across diagnostic groups. Moderate impairment cases were concentrated within the 30%–45% interval, while more severe cases clustered within the 46%–59% interval. These intervals captured more than 90% of observations in the major diagnostic groups. Differences in disability distributions between diagnostic groups were statistically significant (*p* < 0.001). Sensitivity analyses indicated that the identified interval boundaries were recurrent under repeated resampling.

**Conclusions:**

The proposed interval-based approach provides a reproducible, data-driven representation of work disability severity. Rather than demonstrating improved consistency in expert assessment, the findings identify recurring concentration patterns that may provide a quantitative reference for further research on disability assessment. Further external and clinical validation is required before the approach can be considered for implementation in disability assessment practice. These findings are relevant for population-level assessment of work disability and may inform public health monitoring and workforce participation policies.

## 1 Introduction

Assessment of work disability is a fundamental component of occupational health systems, as it directly influences compensation decisions, return-to-work planning, and long-term workforce participation. In many countries, disability evaluation is based on expert judgment, where clinical information is translated into percentage-based estimates of functional impairment. Despite its importance, this process remains vulnerable to variability across assessors, which may reduce consistency and transparency in occupational health decision-making (1, 2).

From an occupational health perspective, work disability reflects not only individual health conditions but also broader patterns of functional limitation across worker populations. Large-scale administrative and epidemiological data provide an opportunity to investigate these patterns and to identify systematic regularities in disability outcomes (3, 4). Such data-driven approaches are increasingly recognized as essential for improving the evidence base of occupational health practice.

Disability assessment is a complex process in which clinical information must be translated into judgments concerning functional limitations and work capacity. Previous research has demonstrated substantial variability in professional judgments during disability assessment, raising concerns regarding inter-rater reliability and reproducibility (5, 6). Sources of variation may include the assessment process itself, the instruments used, and characteristics of the assessor (7). At the same time, occupational disability cannot be understood solely in terms of a medical diagnosis, as work functioning encompasses both work activities and participation and is influenced by individual and contextual factors (8). The International Classification of Functioning, Disability and Health (ICF) has therefore been increasingly used as a framework for describing functioning and disability in vocational rehabilitation and work-related health research (8).

Although standardized approaches and structured instruments have been developed to improve the reliability of disability assessment, evidence indicates that agreement between assessors can remain variable (5, 6). Moreover, a substantial proportion of the existing literature. focuses on the reliability of individual assessment instruments, functional measures, or return-to-work outcomes rather than on the empirical distribution of percentage-based disability ratings in large populations. Consequently, less is known about whether disability severity exhibits recurring concentration patterns within diagnostic groups that can be identified and quantified using large-scale administrative data.

This gap provides a rationale for examining disability severity as an empirical distribution rather than solely as a categorical or expert-determined outcome. In applied epidemiological research, heterogeneous empirical distributions can be characterized by clustering or concentration effects, where observations accumulate within specific value ranges (9). Identifying such concentration zones may provide a complementary quantitative framework for characterizing work disability severity. However, the use of interval-based statistical approaches to characterize the population-level distribution of work disability severity remains insufficiently explored.

The present study aims to develop and evaluate an interval-based statistical approach for identifying recurrent patterns of work disability severity using large-scale medical expert data. Using a national dataset of occupational disability assessments, we examine whether disability percentages exhibit diagnosis-specific concentration patterns and whether these patterns can be supported by statistical inference. By linking empirical distribution analysis with occupational health data, this study provides a quantitative framework for characterizing observed variation in disability severity and establishes a basis for further evaluation of interval-based approaches to disability assessment.

From a public health perspective, work disability represents an important indicator of population health and workforce sustainability. Understanding its distribution patterns may provide insights into broader health inequalities and inform health system planning.

In this context, disability severity can be interpreted as a population-level outcome reflecting functional health status and its impact on workforce participation.

## 2 Methods

### 2.1 Study design and data source

This study employed a retrospective observational design based on administrative data provided by the Center for Labor Resources Development (CLRD) of the Republic of Kazakhstan. For the quantitative and structural analysis of the distribution of work disability severity, the CLRD provided a dataset containing records for 4,863 persons with disabilities for whom a degree of loss of occupational capacity had been established between January 1, 2020 and December 31, 2024. The dataset was provided with a regional breakdown, allowing the distribution of disability assessments to be examined across regions of the Republic of Kazakhstan.

The study population comprised persons with disabilities for whom a degree of loss of occupational capacity had been established and who were represented in the administrative dataset provided by the CLRD during the study period. Inclusion in the analytical dataset was based on the presence of an established degree of loss of occupational capacity. The dataset included ICD-10 diagnostic codes, the percentage of loss of occupational capacity, and selected demographic characteristics, including age and sex.

The data were provided in anonymized form and contained no personally identifiable information. The present analysis was based on routinely collected administrative records and did not involve additional clinical assessment or intervention by the researchers (4, 10, 11).

The analytical strategy was designed to address the primary objective of the study, namely, to identify and quantify recurring concentration patterns in disability severity rather than to establish the clinical validity of disability ratings. The analytical approach was therefore focused on characterizing the observed distribution of disability severity and assessing the reproducibility of identified concentration patterns within the study dataset.

The dataset was obtained from the Center for Labor Resources Development (CLRD) and consisted of administrative records of persons for whom an official degree of loss of occupational capacity had been established during the study period. The present study did not have access to the technical documentation of the underlying information system; therefore, system-level details regarding its architecture and complete national coverage were not independently verified.

### 2.2 Data preprocessing and inclusion criteria

A structured data preprocessing procedure was applied to define the analytical population and ensure consistency of the variables used in the analysis. The initial dataset comprised 4,863 administrative records. Records with missing values in variables required for the primary analysis were excluded. No imputation of missing values was performed. The number of records excluded because of missing data was recorded before application of the remaining eligibility and exclusion criteria. Disability severity values were restricted to the valid range of 0%–100%.

The primary analytical population was defined as persons with chronic occupational and work-related diseases for whom a degree of loss of occupational capacity had been established. Accordingly, acute traumatic injuries were not included in the primary analysis because they fell outside the predefined target population of chronic occupational and work-related conditions. Diagnostic categories represented by single observations or extremely small numbers of observations were excluded from the analysis because their sample sizes were insufficient for reliable estimation of concentration intervals. The remaining 161 ICD-10 codes were aggregated into five broader categories of chronic occupational and work-related diseases for the primary analysis. Neuropsychiatric conditions requiring specialized assessment frameworks were also excluded because their assessment may involve functional and clinical criteria that are not directly comparable with the percentage-based impairment measure analyzed in the present study.

Pathologies for which the percentage-based loss-of-occupational-capacity measure was not applicable were likewise excluded. These criteria were applied to define a clinically and analytically coherent study population rather than to select observations according to the observed statistical results. After application of the eligibility and exclusion criteria, the final analytical dataset comprised 2,065 observations representing 161 ICD-10 diagnostic codes.

Because these criteria restrict the analytical population, the possibility of selection bias cannot be excluded. Therefore, the findings are interpreted as describing the analyzed population of chronic occupational and work-related conditions and should not be generalized to all causes of work disability without external validation.

The final sample size was determined by the availability of eligible administrative records during the study period rather than by an *a priori* power calculation. All records meeting the predefined eligibility criteria were retained for the primary analysis. Diagnostic groups with extremely small numbers of observations were excluded because their sample sizes were insufficient for reliable estimation of concentration intervals.

### 2.3 Classification of diagnostic groups

Diagnoses were classified according to the International Classification of Diseases, 10th Revision (ICD-10), which is widely used in epidemiological and occupational health research (11).

For analytical purposes, individual diagnoses were aggregated into broader diagnostic categories to ensure sufficient sample size and to reduce variability associated with rare conditions. Such aggregation is commonly applied in population-based studies to enhance statistical stability and comparability of results (9, 12).

### 2.4 Statistical analysis

The primary objective of the statistical analysis was to identify and quantify recurring concentration patterns of disability severity within the predefined diagnostic groups. Disability percentage values were treated as empirical observations rather than as normally distributed measurements. Descriptive statistics, histograms, and kernel density estimation were used to characterize the shape and distribution of disability severity (13).

For each diagnostic group, a concentration function was defined as the proportion of observations falling within a specified interval. An interval was considered a dominant concentration interval when it contained at least 80% of observations within the respective diagnostic group. This threshold was used as an operational criterion to identify intervals representing the predominant concentration of disability severity rather than as a clinical cut-off. Confidence intervals for the estimated proportions were calculated using binomial proportion methods.

Differences between empirical distributions were assessed using the Mann–Whitney U test and the Kolmogorov–Smirnov test because the disability percentage data did not require the assumption of normality. A two-sided *p*-value <0.05 was considered statistically significant.

The statistical procedures were selected to address complementary aspects of the research question: descriptive and density-based methods characterized the empirical distribution; concentration functions quantified the proportion of observations within candidate intervals; confidence intervals quantified uncertainty around concentration estimates; non-parametric tests assessed distributional differences between diagnostic groups; and sensitivity analyses examined the reproducibility of the identified interval patterns.

### 2.5 Sensitivity analysis

To assess the stability of the identified interval structures, several sensitivity analyses were conducted (14). Alternative interval widths were examined to determine whether the observed concentration patterns persisted under different interval specifications. The dataset was also randomly divided into training and validation subsets to examine the reproducibility of the identified patterns. In addition, a Monte Carlo resampling procedure comprising 1,736 resampling iterations was performed to assess the frequency with which the predefined intervals were reproduced as dominant concentration intervals. The proportion of simulations in which each interval was reproduced was used as the measure of interval stability.

### 2.6 Statistical software

All statistical analyses were performed using standard statistical computing environments, including Python and R. Data processing, visualization, and modeling were conducted using widely used libraries for statistical analysis.

The analysis was conducted from a population health perspective using large-scale administrative data.

The study was conducted and reported in accordance with the STROBE guidelines for observational studies.

## 3 Results

### 3.1 Sample characteristics

After applying the predefined inclusion and exclusion criteria, the final analytic dataset included 2,065 observations representing occupational disability assessments across 161 ICD-10 diagnostic categories.

After applying the predefined inclusion and exclusion criteria, the final analytical dataset included 2,065 observations covering 161 ICD-10 codes. The distribution of cases across diagnostic groups was heterogeneous, with the largest contributions from musculoskeletal and respiratory conditions (Table 1).

**Table 1:** Distribution of observations across major diagnostic groups.

Diagnostic group	Number of cases	Share (%)
Radiculopathy with complications	841	40.73
Respiratory diseases with complications	567	27.46
Radiculopathy without complications	285	13.80
Respiratory diseases without complications	210	10.17
Chronic intoxication	162	7.84
Total	2,065	100

Disability severity values ranged approximately from 25% to 60%, with visible clustering patterns across diagnostic groups.

### 3.2 Distribution of disability severity

Exploratory analysis revealed a non-uniform distribution of disability percentage values, with two dominant concentration zones. The moderate impairment interval was 30%–45%, whereas the more severe impairment interval was 46%–59% (Figure 1). Kernel density estimation provided a complementary representation of the observed distribution (Figure 2).

**Figure 1:**
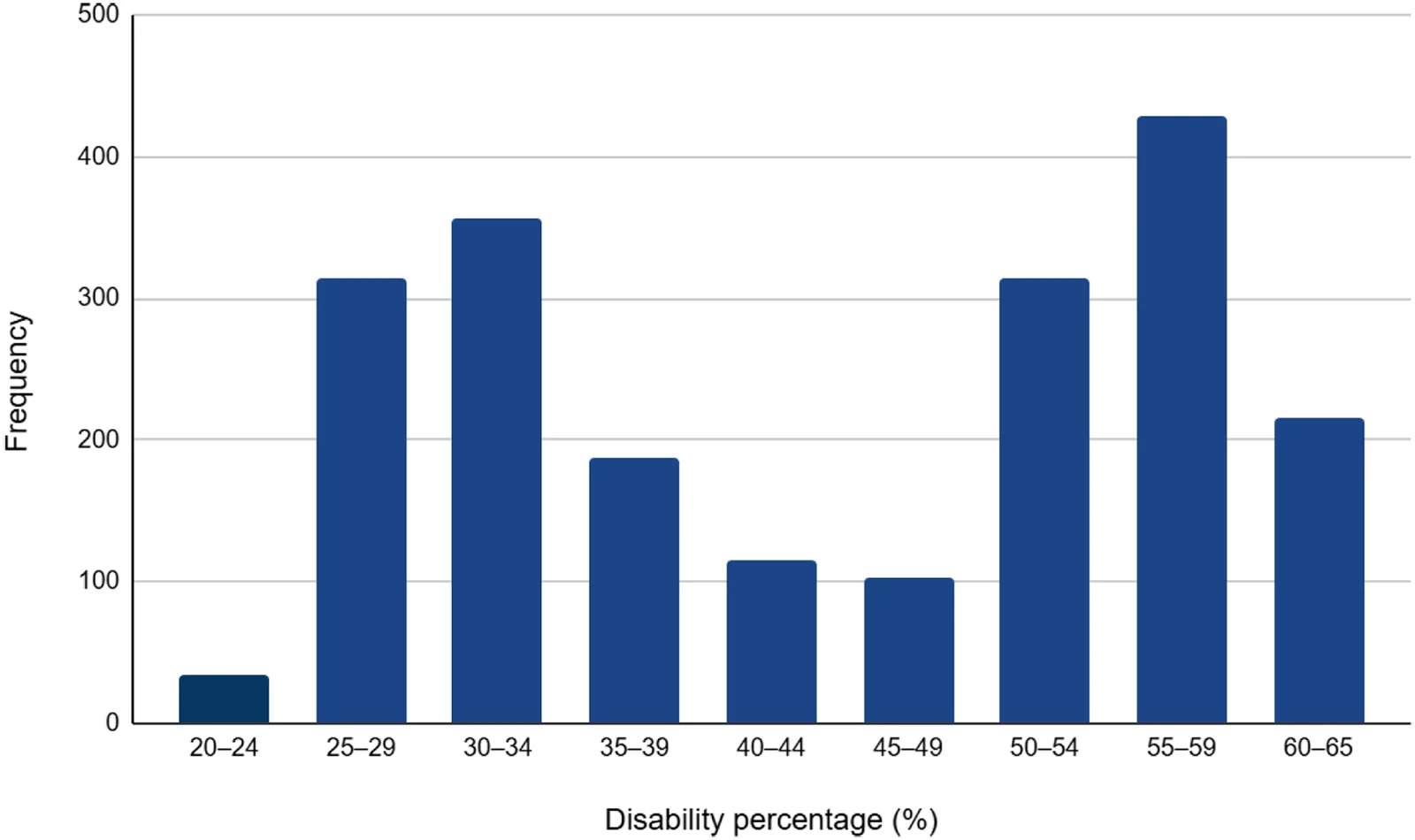
Histogram of disability percentage distribution using 5% interval bins.

**Figure 2:**
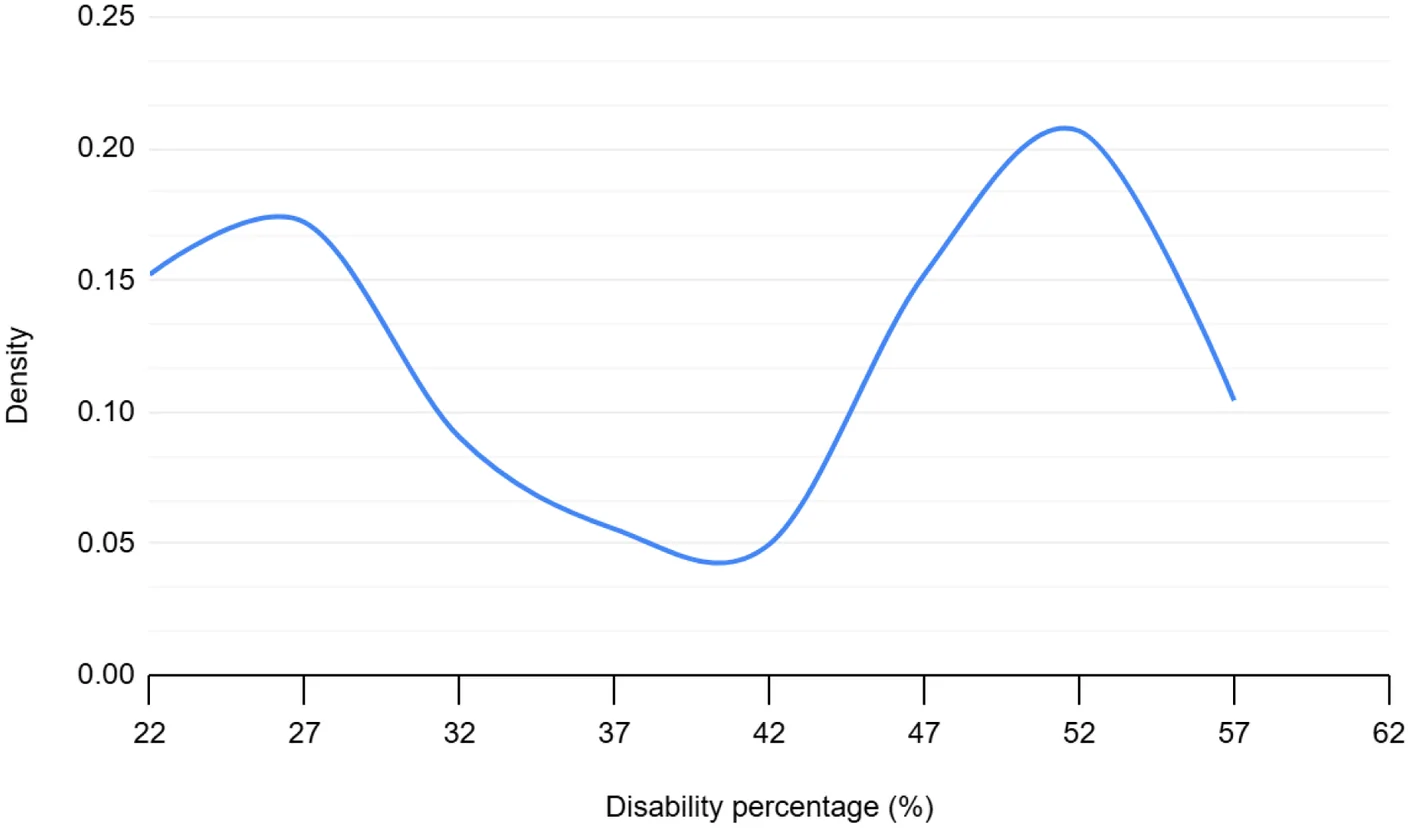
Kernel density estimate of disability percentage distribution.

Statistical comparison between diagnostic groups with and without complications showed significant differences in disability distributions. The Mann–Whitney U test indicated a statistically significant difference (*p* < 0.001), and the Kolmogorov–Smirnov test likewise indicated that the empirical distributions differed (*p* < 0.001).

### 3.3 Dominant intervals of disability severity

For diagnostic groups involving complications, disability severity was concentrated within the 46%–59% interval.

For diagnostic groups involving complications, the 46%–59% interval was dominant, with concentration estimates exceeding 95% in the groups shown in Table 2. The dominant intervals of disability severity across diagnostic groups are presented in Table 2.

**Table 2:** Dominant disability intervals across diagnostic groups.

Diagnostic group	Dominant interval	Concentration (%)	95% CI
Radiculopathy with complications	46%–59%	95.01	93.53–96.48
Respiratory diseases with complications	46%–59%	97.48	94.66–100
Radiculopathy without complications	30%–45%	90.63	83.48–97.77
Respiratory diseases without complications	30%–45%	91.04	86.21–95.88
Chronic intoxication	30%–45%	89.38	84.60–94.15

### 3.4 Moderate impairment groups

For diagnostic groups without complications, the dominant concentration interval was 30%–45%, with concentration estimates of approximately 89%–91% across the groups shown in Table 2.

The proportion of observations within dominant intervals was significantly higher in severe impairment groups compared with moderate impairment groups (two-proportion test, *p* < 0.001).

### 3.5 Distributional characteristics

Across diagnostic groups, empirical distributions exhibited recurring statistical features, including unimodal clustering within strata, asymmetric tails, and a limited number of extreme values.

These findings are consistent with known properties of empirical health-related data distributions, which often exhibit clustering and non-normal behavior (13).

### 3.6 Robustness analysis

To assess the stability of the identified intervals, a Monte Carlo resampling procedure comprising 1,736 iterations was conducted. The moderate impairment interval (30%–45%) was reproduced in 94.7% of simulations, while the severe impairment interval (46%–59%) was reproduced in 96.3% of simulations. These results indicate that the identified interval boundaries were frequently reproduced under repeated resampling.

For each resampling iteration, a bootstrap sample of the same size as the original analytical dataset (*n* = 2,065) was generated with replacement. The predefined concentration intervals (30%–45% and 46%–59%) were then evaluated in each resampled dataset using the same concentration criterion as in the primary analysis. An interval was considered reproduced when it remained the dominant concentration interval under the predefined criterion. A total of 1,736 resampling iterations were performed, and the proportion of iterations in which each interval was reproduced was calculated.

## 4 Discussion

### 4.1 Principal findings

The results indicate that work disability severity is not uniformly distributed across the examined diagnostic groups and exhibits recurring concentration intervals. Two dominant intervals were identified: 30%–45% for moderate impairment and 46%–59% for more severe impairment. These intervals captured more than 90% of observations within the major diagnostic groups and were frequently reproduced under repeated resampling. However, the study does not establish whether these patterns arise from clinical characteristics, assessment practices, regulatory criteria, diagnostic classification, temporal or regional differences, or other factors.

In addition, the observed bimodal distribution provides a rationale for future investigation using formal tests of multimodality, such as the dip test, and model-based approaches such as finite mixture modelling and latent class analysis (15–17). Such approaches could help determine whether the observed distribution reflects statistically distinguishable subgroups or other forms of population heterogeneity. However, these hypotheses were not formally tested in the present study.

### 4.2 Interpretation in the context of occupational health

From an occupational health perspective, the findings indicate that the observed disability outcomes may reflect not only individual clinical conditions but also system-level factors related to how functional impairment is assessed and operationalized.

Previous studies have highlighted substantial inter-rater variability in disability evaluation, raising concerns regarding consistency and reproducibility (3, 5, 7, 18, 19). The present results extend this literature by showing that observed disability ratings were concentrated within recurring statistical intervals.

The observed concentration patterns may reflect multiple factors, including clinical characteristics, assessment practices, regulatory criteria, diagnostic classification, and institutional context. However, the present study was not designed to disentangle these potential mechanisms. Therefore, the identified intervals should be interpreted as empirical features of the observed administrative data rather than as evidence of shared decision-making behavior among experts.

### 4.3 Methodological implications

The proposed interval-based approach provides a distribution-informed description of observed disability severity rather than a replacement for clinical or expert assessment. By identifying recurrent concentration intervals in administrative data, the approach may provide a quantitative reference for further investigation of variability in disability assessment. Whether such intervals can reduce subjectivity, improve inter-rater consistency, or enhance decision-making requires independent clinical and prospective evaluation.

Unlike conventional methods that rely on predefined thresholds or expert judgment alone, the present approach uses empirical data to identify dominant probability regions of disability severity. This aligns with modern trends in applied epidemiology and statistical modeling, where heterogeneous data are analyzed through distributional structures and clustering effects.

Furthermore, the bimodal pattern observed in this study suggests that disability severity may be conceptualized as a mixture of underlying probabilistic components, corresponding to different levels of functional impairment. This provides a rationale for future investigation using mixture modeling or latent class analysis to examine whether the observed concentration patterns correspond to distinct underlying components.

### 4.4 Implications for occupational health practice and policy

The findings have important implications for both practice and policy in occupational health systems.

First, the identification of recurring disability severity intervals provides a data-driven reference framework for further investigation of variability in disability assessment across diagnostic groups, evaluators, and institutions. However, the present study did not assess whether their application is associated with differences in disability assessment outcomes across demographic, geographic, diagnostic, or institutional subgroups.

Second, the interval-based approach may provide an additional quantitative perspective for the development and evaluation of disability assessment frameworks. Its potential relevance to compensation decisions and return-to-work policies should be evaluated in conjunction with clinical, functional, and occupational information rather than used as a standalone basis for decision-making.

From a policy perspective, the observed concentration patterns may be useful for identifying areas requiring further investigation of variation in disability assessment across regions, institutions, or diagnostic groups. However, the present study does not provide evidence that the proposed approach can harmonize assessment practices or reduce regional or institutional variation.

The use of large-scale administrative data also illustrates the potential of routinely collected information for population-level characterization of work disability. The identified intervals may provide descriptive indicators for monitoring the distribution of disability severity over time, provided that their stability and comparability are confirmed in longitudinal and external datasets.

Finally, the implications of the findings for equity, fairness, and access to social protection remain to be investigated. Future research should examine whether interval-based approaches produce comparable results across demographic, geographic, diagnostic, and institutional subgroups and whether their application is associated with more equitable disability assessment outcomes.

### 4.5 Strengths and limitations

This study has several strengths. It is based on a large real-world dataset covering multiple diagnostic categories, which enhances the robustness and practical relevance of the findings. The use of multiple statistical techniques, including distribution analysis, inferential testing, and Monte Carlo resampling, provides complementary statistical evidence for the observed concentration patterns.

However, several limitations should be acknowledged. The analysis is based on data from a single national administrative dataset, and no independent external dataset was available for validation. Therefore, the generalizability and transportability of the identified intervals to other occupational health systems, populations, or regulatory settings cannot be established from the present study. The retrospective design does not allow causal inference, and potential variability in diagnostic coding or expert evaluation procedures may have influenced the observed patterns. In addition, the exclusion criteria may have introduced selection bias, and the findings should therefore be interpreted as applicable to the analyzed population rather than to all forms of work disability. The study also does not establish clinical validity or improvements in inter-rater reliability, which require independent clinical assessment and prospective external validation.

Several potential sources of bias should also be considered when interpreting the observed concentration patterns. First, the administrative dataset may contain regional variation in assessment practices, diagnostic coding, or institutional procedures, which could contribute to differences in the observed distributions. Second, potential classification or coding errors cannot be excluded because the analysis relied on routinely recorded administrative diagnoses. Third, the study period covered 2020–2024, during which changes in regulatory requirements, assessment practices, or public policies may have influenced the distribution of disability ratings. The present study was not designed to disentangle these temporal, regional, or policy-related effects. Therefore, the identified intervals should be interpreted as empirical characteristics of the observed administrative dataset rather than as invariant features of disability assessment across regions, time periods, or policy settings.

In addition, the administrative dataset did not contain sufficient information on specific occupational characteristics, such as job demands, occupational exposures, workplace conditions, employment history, or other work-related contextual factors. Therefore, the study could not directly assess how occupational characteristics may have contributed to the observed patterns of disability severity.

### 4.6 Future research

Future research should focus on external and clinical validation of the proposed interval-based framework using data from different countries and occupational health systems to assess its generalizability and transferability.

Further studies should examine the identified intervals alongside clinical, functional, and occupational assessment measures to determine their relationship with independently assessed disability severity and functional limitations. Prospective studies could also evaluate whether the use of interval-based information influences inter-rater variability or decision-making in disability assessment.

In addition, extending the current framework using mixture models or latent variable approaches may provide further insight into the underlying structure of disability severity and help determine whether the observed concentration patterns correspond to distinct latent components.

Future research should also examine the effects of diagnostic classification, regional and institutional differences, temporal changes, and relevant policy or regulatory factors on the observed distribution of disability severity. These analyses would help determine the extent to which the identified intervals are reproducible across settings and whether they have practical value for disability assessment.

## 5 Conclusions

The findings indicate that work disability severity exhibits recurring concentration patterns across the examined diagnostic groups. The identified intervals of 30%–45% and 46%–59% captured the majority of observations in the major diagnostic groups and were frequently reproduced under repeated resampling. These findings provide a statistically grounded description of the observed distribution of work disability severity.

The proposed interval-based framework may provide a quantitative reference for further research on disability assessment and occupational health. However, the present study does not establish clinical validity, causal mechanisms, or improvements in assessment consistency. Whether the identified intervals can contribute to more consistent disability assessment or support decision-making requires independent clinical, external, and prospective validation.

The findings may inform further research on disability assessment and workforce participation, while their applicability to other populations, occupational health systems, and regulatory settings remains to be established.

## Data Availability

The raw data supporting the conclusions of this article will be made available by the authors, without undue reservation.
